# Intermediates: ubiquitous species on folding energy landscapes?

**DOI:** 10.1016/j.sbi.2007.01.003

**Published:** 2007-02

**Authors:** David J Brockwell, Sheena E Radford

**Affiliations:** Astbury Centre for Structural Molecular Biology, Institute of Molecular and Cellular Biology, University of Leeds, Leeds LS2 9JT, UK

## Abstract

Although intermediates have long been recognised as fascinating species that form during the folding of large proteins, the role that intermediates play in the folding of small, single-domain proteins has been widely debated. Recent discoveries using new, sensitive methods of detection and studies combining simulation and experiment have now converged on a common vision for folding, involving intermediates as ubiquitous stepping stones en route to the native state. The results suggest that the folding energy landscapes of even the smallest proteins possess significant ruggedness in which intermediates stabilized by both native and non-native interactions are common features.

## Introduction

One of the major advances in studies of protein folding over the past 15 years was the discovery that, in the absence of complicating factors such as proline isomerization, most small proteins (<100 amino acids) fold to their native structure in a cooperative two-state transition [1]. In such (un)folding pathways, only the native and denatured states are significantly populated. The simplicity of the folding mechanism of this class of protein has had enormous impact on the development of our understanding of folding, focusing attention on the structures of transition state ensembles [2], driving attempts to capture the essential features of folding using computer-based models [3], and underpinning attempts to delineate mathematical relationships between protein sequence, structure and folding rate constants [4]. However, two-state folding not only precludes a detailed view of different stages in folding pathways, but also masks the underlying complexity of a process that involves finding a single structure, stabilized by a myriad of specific interatomic interactions, from the astronomical number of possible alternatives available. For these reasons, the characterization of intermediates (metastable states that are transiently populated on the folding free energy landscape) has proved attractive to experimentalists and theoreticians alike [5].

It is generally accepted that proteins greater than 100 amino acids in size fold via one or more intermediate states that act as stepping stones to the native state. However, the role or even the presence of metastable states during the folding of smaller proteins has proved highly controversial [6], even though two-state behaviour does not preclude the existence of high-energy states along the folding pathway [7]. What role, then, do intermediates play in folding? Are they off-pathway species that might be the progenitors of human disease, or misfolded states that slow the rate of folding [8]? Alternatively, could intermediates play a role in correct folding, even for proteins that apparently fold efficiently without them?

Here, we summarize recent advances in experimental data that address these questions and the insights gained. We also discuss the emerging view that potentially all proteins fold via intermediate states.

## Detecting intermediates

### Effect on kinetic chevron plots

**Figure 1:**
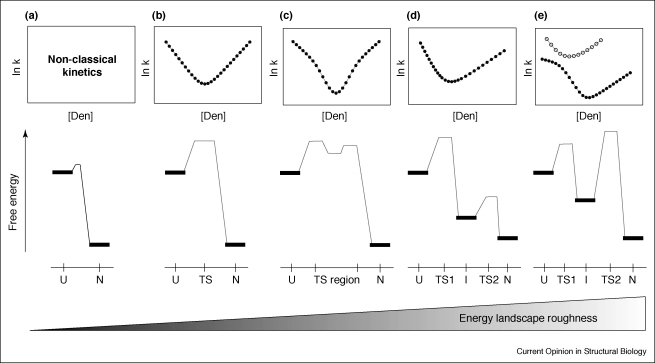
Example chevron plots (top) and free energy diagrams (bottom) for different folding scenarios. Each chevron plot shows the denaturant ([Den]) dependence of the observed rate constant (ln k). **(a)** Barrier-less folding. **(b)** Classical two-state folding, in which intermediates are not populated. **(c)** Folding via a high-energy intermediate. The switch between early and late transition states as a function of denaturant concentration results in non-linearity of the folding and unfolding branches of the chevron plot. **(d)** Population of a late folding intermediate, subsequent to the rate-limiting transition state. Such proteins might display two-state kinetics and the population of intermediates is typically inferred from native state hydrogen exchange experiments. **(e)** Folding via the population of a stable intermediate in the first millisecond of folding. This is revealed by the so-called ‘rollover’ in the folding branch of the chevron plot determined using stopped-flow methods (closed circles). In the case where the intermediate accumulates rapidly during folding, a second chevron plot reflecting the formation and unfolding of the intermediate can be obtained using ultra-rapid mixing experiments (open circles). The positions of the denatured state (U), intermediate state (I), early and late transition states (TS1 and TS2, respectively) and native state (N) are shown on an arbitrary scale.

A powerful method of detecting intermediates formed during folding or unfolding is to analyse the denaturant dependence of the folding and unfolding kinetics using chevron analysis. In the absence of a significant transition state barrier (<2 k_B_T), folding is predicted to occur in a downhill manner [9], ruling out analysis of folding using chevron analysis (Figure 1a). By contrast, the presence of a single transition state barrier on an otherwise smooth energy landscape results in classical V-shaped chevron plots in which the logarithm of both the folding and unfolding rate constants depend linearly on the denaturant concentration (Figure 1b). Such a scenario is indicative of two-state folding, for which chymotrypsin inhibitor 2 (CI2) remains the paradigm example [7]. Deviations from linearity in either branch of the chevron plot (Figure 1c,e) are consistent with the transient population of metastable states on the folding energy landscape. Where no direct experimental evidence can be found for the accumulation of an intermediate during folding (such as the absence of a burst phase change in the signal amplitude), non-linearity in the chevron plot can be interpreted as reflecting either a gradual change in the position of the rate-limiting transition state on the reaction coordinate (Hammond effects), as classically observed for *Thermus thermophilus* ribosomal protein S6 [10], or a denaturant-dependent switch between distinct transition states on a sequential pathway [11]. The latter mechanism necessarily invokes the transient formation of a high-energy intermediate that is on-pathway for folding, as shown in Figure 1c. Kiefhaber and co-workers have fitted the folding and unfolding kinetics of 23 small proteins to the latter model, suggesting that folding via high-energy intermediates is a common occurrence in folding energy landscapes [11,12].

Intermediates can also be formed after the rate-limiting transition state has been traversed. Such species might not be detected using chevron analysis (Figure 1d), unless the intermediate is unusually stable. Such late intermediates can be revealed, however, by measuring conformational fluctuations from the native state using, for example, native state hydrogen exchange or NMR relaxation methods (see [13,14] and reviews published elsewhere in this issue). These techniques have been used to identify hidden intermediate states that occur after the rate-limiting transition state in cytochrome *c* [13], barnase [15], a redesigned apo-cytochrome *b*_562_ [16,17], the third PDZ domain from PSD-95 [18], acyl-coenzyme A binding protein (ACBP) [19^•^] and T4 lysozyme [20]. In addition, recent simulations indicate that the Fyn Src homology 3 (SH3) domain might also unfold via such an intermediate [21]. Native state hydrogen exchange has also been used to identify misfolded off-pathway intermediates in the folding energy landscape of 179-residue apoflavodoxin [22]. From a physiological viewpoint, such species might be important in the control of signalling pathways [23], or might provide a link between the protein folding and aggregation energy landscapes [8,24].

When stable intermediates are formed before the rate-limiting transition state for folding, a distinct rollover in the folding branch of the chevron plot results (Figure 1e). In this scenario, the intermediate accumulates in the first millisecond of folding and the kinetic rollover is accompanied by a change in signal amplitude in the burst phase of stopped-flow experiments. Rapid formation of the populated intermediate can be directly detected using ultra-rapid mixing experiments [6], giving rise to a second chevron plot that describes the folding and unfolding kinetics of the intermediate via an early transition state. Folding via such a mechanism has now been observed for several small proteins, including the small helical bacterial immunity protein Im7 [25], ACBP [26], ubiquitin [6], cytochrome *c* [6] and the human HYPA/FBP11 FF domain [27]. For these proteins, folding occurs over a relatively rough landscape whose energy maxima and minima can be tailored by alterations in sequence, temperature or solvent conditions. As a consequence, relatively minor changes in sequence or folding conditions can result in dramatic changes in kinetic behaviour [28,29]. With careful interpretation, therefore, complexities in the chevron plot can provide a powerful means of detecting roughness in the folding energy landscape, exposing the presence of intermediate species even when they do not accumulate during folding. For those intermediate states that are stable enough to withstand mutational analysis, kinetic analysis enables indirect determination of their structural characteristics, by using φ-analysis [2], for example.

### Rare and transiently populated states

For some proteins, the transient formation of intermediates during folding (or unfolding) might elude detection using stopped-flow techniques that are capable of measuring folding events on millisecond timescales. For such species, increasing the temporal resolution of the method of initiating folding or increasing the sensitivity of the detection method can reveal the presence of such species. For example, the small four-helix protein ACBP was previously considered to fold with two-state kinetics; however, an early folding intermediate has now been observed for this protein by monitoring folding using ultra-rapid mixing methods in combination with detection using Förster resonance energy transfer (FRET) [26]. Fluorescence correlation spectroscopy is another powerful technique that has not been used widely in the context of protein folding. Using this approach, Neuweiler *et al.* [30^•^] measured the folding kinetics of a 20-residue ‘Trp cage’ labelled with an extrinsic fluorophore, revealing novel and hitherto undetected species populated at low denaturant concentrations, consistent with the rapid formation of a partly folded, collapsed state early during folding. Such a species is not detected at high denaturant concentrations, or in a single isoleucine to glycine mutant, suggesting that, even for this tiny protein, collapse to a structured state initiates the folding reaction.

Other exciting developments in our experimental capabilities for measuring protein folding reactions include the use of NMR relaxation measurements such as off-resonance ^15^N rotating frame [31] or relaxation dispersion [14] methods, which are capable of detecting non-native species when their population is exceedingly low (down to ∼0.5%). The former method has been used to characterize an intermediate of the small 67-residue villin headpiece [32]. In the relaxation dispersion method, the degree of dispersion is governed by the population of each species, the difference in chemical shift of each resonance in each state and the rates of their interconversion [33]. The information content of these experiments, especially when combined with ^13^C, ^2^H and ^15^N labelling, is remarkably rich, and can provide detailed structural, kinetic and thermodynamic information about rarely populated states at the level of individual atoms (see [34,35^•^] and reviews by Mittag and Forman-Kay, and Vendruscolo elsewhere in this issue). Such experiments can be used as a rigorous test of whether a protein folds with a two-state transition (at least on the millisecond timescales sensitive to these experiments) or whether rare, partially folded states are populated en route to the native conformation. Thus, whereas for cold shock protein B (CspB) there is excellent agreement between the (un)folding rate constants measured by relaxation dispersion NMR and those obtained from fluorescence stopped-flow measurements [36], similar studies on other well-characterized small model proteins, such as ACBP [19^•^] and Fyn SH3 domains [34], have demonstrated that these small, single-domain proteins fold via rare intermediate states.

As the sensitivity and resolution of our experimental arsenal has increased, the richness of information about folding landscapes has increased in parallel, offering new and exciting opportunities to describe the search for the native state in structural, kinetic and thermodynamic detail.

## Two-state or three-state folding? A unifying view

Although the classification of proteins into those that fold with a two- or three-state transition provides a useful description of the kinetic folding mechanism, recent studies of the folding mechanisms of protein homologues suggest that the structural mechanism of folding might be conserved, even if the kinetic folding mechanism is different. For example, the four-helix bacterial immunity proteins Im9 and Im7 have ∼80% sequence similarity and almost identical native structures; however, they fold at pH 7.0, 10 °C with two- and three-state transitions, respectively [37]. Nonetheless, the rate-limiting transition state for both proteins is a similar three-helix species, implying that the structural mechanism of folding is conserved [38]. By comparing the folding of different proteins with the same topology, a major determinant of protein folding rates, the contact order [4], is mostly unperturbed, whereas factors thought to be important in determining the stability of transient metastable states, such as secondary structure propensity and local hydrophobicity, might vary significantly. Changing these factors thus modulates the stability of metastable states along the folding pathway and thus the complexity of the observed kinetics.

For Im9, increasing the helix propensity or hydrophobicity of specific regions, altering its sequence to incorporate just three residues from Im7, or changing the pH switches the folding mechanism of this protein from two to three state, by specifically stabilizing an intermediate common to both folding pathways such that it becomes visibly populated during folding [28,39,40]. Similar switches, including three state to two state and *vice versa*, have been achieved for other proteins [29,41]. Thus, for some proteins, small changes in sequence can result in gross changes in kinetic mechanism while the structural mechanism of folding remains conserved. In other cases, tailoring the stability of different structural regions or altering their connectivity by the creation of circular permutants can alter the order of formation of structural elements during folding without perturbing the kinetic folding mechanism [42,43^••^,44,45].

Fersht and collaborators [46] have also used the study of protein families, including both experimental and simulation methods, to investigate the folding landscape of four members of the homeodomain superfamily. Three of these small three-helix-bundle proteins, hTRF1 (human telomeric repeat binding factor 1), hRAP1 (human repressor-activator protein 1) and c-Myb (third repeat of the mouse cellular myeloblastis DNA-binding domain) fold in a two-state manner, whereas En-HD (engrailed homeodomain) folds via an intermediate [46]. En-HD was found to have a high α-helical propensity compared with its homologues in the region spanning helices I and II, possibly explaining the unusually high stability of the intermediate in this case. Surprisingly, and apparently in contradiction with the experimental results, molecular dynamics simulations of the unfolding of these proteins suggested that both En-HD and c-Myb fold via an intermediate state, with hRAP1 and hTRF1 folding in a two-state manner. Increasing the propensity to form helix II and the helix-turn-helix motif of c-Myb by deleting a proline residue in this loop resulted in a switch from two- to three-state folding [47^•^], demonstrating the power of combining simulation and experiment in unpicking the intricacies of protein folding mechanisms (see also [48]). In addition, they serve as an important reminder of the multidimensionality of the folding energy landscape, in which only small changes in sequence can cause apparently dramatic changes in the kinetic mechanism by exposing new features of its rugged surface [47^•^].

Together with the results for other proteins highlighted above, these data reveal a new unifying understanding of the relationship between the conservation of sequence, the stability of intermediates and the structural mechanism of folding [46], suggesting that intermediates may be formed generically during folding, even for the simplest proteins [49], and that single mutations may alter the structural properties of the intermediates formed [43^••^,50^•^]. This opens up the challenge to further develop methods to detect these states and to characterize them structurally, so that insights into the structural mechanism of folding for even the smallest proteins can be revealed.

## Effects on folding: breaks or accelerators?

The results discussed above suggest that the folding energy landscapes of even the simplest proteins contain significant ruggedness. This may result from the evolutionary pressure for function over folding, such that the ideal smooth folding energy landscape is compromised by the evolutionary pressure to evolve function. This then raises the question of whether energetic frustration is beneficial or detrimental to folding. In the case of the Pin1 WW domain, the answer is clear: replacement of a six-residue loop that is essential for function with shorter sequences with enhanced propensity to form a β turn increases the folding rate by almost an order of magnitude [51]. Similar results have been observed for the Fyn SH3 domain [52]. These data demonstrate that the evolution of the sequence for functional purposes has compromised both the speed of folding and the native state stability. This energetic frustration might be caused by the formation of non-native interactions involving residues selected for functional purposes, or by the incorporation of residues needed to avoid aggregation, or both.

Ruggedness in the landscape might not always slow folding [53]. In one recent example, destabilization of the helical intermediate of a model Trp cage miniprotein switched the folding of the protein from three to two state and significantly slowed folding [30^•^]. In some proteins, the presence of high-energy intermediates along a folding pathway might also accelerate folding by breaking the configurational search into a series of smaller problems. If these minima in the energy landscape become too deep, however, folding will be slowed by the introduction of kinetic traps, increasing the likelihood of off-pathway events such as protein aggregation [8]. It is interesting in this regard that redesigning the core of apomyoglobin by mutating all of the isoleucine and valine residues to leucine, or by randomly shuffling 12 of the 35 residues that comprise the hydrophobic core of this protein, stabilized its folding intermediate [54]. On the basis of these results, the author suggests that evolution has selected the sequence of the hydrophobic core of this protein so as to destabilize its partially folded state and that this effect may have been important in the molecular evolution of globular proteins [54]. Further experiments are now needed to test whether this is the case for other protein sequences.

## Native versus non-native interactions in folding intermediates

A major change in our appreciation of partially folded states that has emerged in the past few years has been the realisation that folding does not simply occur by the formation of increasing numbers of native interactions, but that non-native interactions can also play an important role in determining folding mechanisms and folding rates. Recent evidence for this assertion has come from both experimental and theoretical studies, involving proteins with different folds [35^•^,52,55,56,57^••^]. For example, non-native interactions, alongside native contacts, have been shown to be involved in stabilizing the unfolded states of proteins in the absence of denaturant, demonstrating that a large part of the conformational search for the native state can be solved during the very initial events of folding [58^••^,59]. Mutational analysis of apomyoglobin has shown that truncation of residues in helices G and H that make no contact in the native state results in destabilization of its burst phase intermediate, consistent with this species adopting a native-like fold stabilized by non-native packing contacts between these helices [56]. A similar scenario is observed in the intermediates formed during the folding of several other helical proteins. Thus, the intermediates formed during the folding of En-HD [58^••^], apo-cytochrome *b*_562_ [57^••^] and Im7 [25] are all stabilized by the formation of both native and non-native interactions, so as to optimize burial of hydrophobic surface area in their partially folded forms.

The formation of non-native interactions during folding is not exclusive to all-α-helical proteins: non-native packing of hydrophobic sidechains has also been shown to play an important role in stabilizing the intermediates formed during the folding of α-lactalbumin [60], lysozyme [61], SH3 domains [35^•^] and CD2.d1 [55]. Non-native contacts thus appear to be a common feature of folding energy landscapes, playing a role in the folding of proteins spanning all structural classes. As long as kinetic traps do not develop, the formation of non-native contacts might increase the rate of folding by minimizing the search for the native state to fewer dimensions [62].

The discovery that many proteins might form transient non-native contacts during folding has important implications. For example, because non-native interactions are not considered in simple Gö-type models, their presence raises questions about how well such potentials can describe the energy landscapes of proteins that fold via the formation of substantial numbers of non-native contacts. The realisation that non-native contacts form during folding also raises questions as to how we might best interpret the results of φ-value analyses and native state hydrogen exchange experiments that use the native state as the reference point, and how we can describe and appreciate the structures of such states. We also need to be careful about how we define non-native contacts, as they could involve a range of different scenarios, from the slight re-organization of sidechain packing in a structure that is otherwise highly native-like, to the formation of interactions far from those that define the native structure.

## Visualizing intermediates through experiments and simulation

Given that partially folded states appear to be ubiquitous features on folding energy landscapes, it is of paramount importance to determine their structural properties, because they offer powerful opportunities to elucidate the structural mechanisms of folding in detail. However, the rarity and transient nature of intermediates usually require the employment of spectroscopic techniques that are amenable to rapid timescales for their detection; yet such techniques provide little detail at the molecular level.

The potential of NMR to provide atom-specific detail about proteins in solution places this technique amongst the most powerful for the elucidation of the structure of non-native states. A plethora of different NMR approaches, including both direct and indirect methods, have now been applied to studies of protein folding [33]. For example, introduction of mutations or alteration of the solution conditions to specifically destabilize the native state allows transient folding intermediates to be trapped at equilibrium and directly analysed using NMR. For some proteins, the intermediates trapped in this manner are well defined, giving rise to NMR spectra that enable detailed structural analysis using approaches developed for native structures [20,57^••^,58^••^]; in other cases, however, the spectra display substantial chemical exchange broadening, preventing direct analysis [63]. Given their dynamic nature and potential conformational heterogeneity, intermediates present experimental challenges concerning how to determine, define and portray their structures. Even for the latter cases, however, structural elucidation is now possible using restrained molecular dynamics simulations. In this approach, coarse-grained parameters such as residue-specific φ-values [64], hydrogen exchange protection factors [65^••^] or chemical shifts [34] are used as restraints, and molecular dynamics simulations are used to derive an ensemble of structures that best describe the experimental parameters. For example, using molecular dynamics simulations restrained by experimentally derived hydrogen exchange protection factors, a structural ensemble representing the intermediate ensemble of Im7 has recently been generated, revealing that this species has a native-like topology and involves an array of structures in which its three helices are packed in slightly different arrangements [65^••^].

These calculations are powerful in that they open the door to images of partially folded states in all-atom detail, revealing precise details about the identity of residues making native and non-native contacts in these states, and stimulating further experiments to validate the predictions made.

## Conclusions and challenges ahead

Taken together, the data discussed above demonstrate that intermediates, stabilized by both native and non-native interactions, are commonly formed en route to the native state. By increasing the power of our experimental capabilities and by combining the powers of theory, simulation and experiment, today's views of folding involve a landscape that contains significant ruggedness, even for the smallest and simplest proteins known.

These observations pose opportunities and challenges for the years ahead. First, we need to develop better methods of elucidating the identity (native or non-native) of interactions formed during folding and of tracking their exchange as the native state develops. Second, the observation that only a handful of the original list [1] of two-state proteins remain for which intermediates have not been detected poses questions about whether the population of intermediates is a pre-requisite for rapid and efficient folding, or results from the stochastic nature of the evolution of structure during folding reactions. The ability to detect intermediates in simple, small proteins, including those that fold on rapid timescales, provides exciting opportunities to develop new insights into the intricacies of folding mechanisms by combining the power of experiments and simulation, as a prelude to tackling larger proteins for which the folding energy landscape will be more complex and more rugged. The commonality of intermediates during folding also rationalizes the need for molecular chaperones in all forms of life, so as to avoid problems arising from potential aggregation of these states [66], suggesting the co-evolution of protein sequences and their folding assistants. Finally, as our knowledge of the structure of intermediates advances, we might gain a better appreciation of the differences between intermediates that are beneficial to folding, those that open the door to aggregation events and those that play an important functional role, providing a platform for the design of new proteins that fold and function efficiently, while avoiding species that could prove harmful to life. Rather than being rare, complicating features of folding energy landscapes, intermediates appear to be a common feature of folding, possibly of all natural protein sequences.

## References and recommended reading

Papers of particular interest, published within the annual period of review, have been highlighted as:

• of special interest
•• of outstanding interest
